# Unraveling bacterial stress responses: implications for next-generation antimicrobial solutions

**DOI:** 10.1007/s11274-024-04090-z

**Published:** 2024-07-29

**Authors:** Fatma Gizem Avci

**Affiliations:** 1 Department of Bioengineering, Faculty of Engineering and Natural Sciences, Üsküdar University, Istanbul, Türkiye; 2 Genetics of Prokaryotes, Faculty of Biology and Center for Biotechnology (CeBiTec), Bielefeld University, Bielefeld, Germany

**Keywords:** Antimicrobial resistance, Bacterial stress response, Drug target, Pathogens, Reporter metabolite

## Abstract

The accelerated spread of antimicrobial-resistant bacteria has caused a serious health problem and rendered antimicrobial treatments ineffective. Innovative approaches are crucial to overcome the health threat posed by resistant pathogens and prevent the emergence of untreatable infections. Triggering stress responses in bacteria can diminish susceptibility to various antimicrobials by inducing resistance mechanisms. Therefore, a thorough understanding of stress response control, especially in relation to antimicrobial resistance, offers valuable perspectives for innovative and efficient therapeutic approaches to combat antimicrobial resistance. The aim of this study was to evaluate the stress responses of 8 different bacteria by analyzing reporter metabolites, around which significant alterations were observed, using a pathway-driven computational approach. For this purpose, the transcriptomic data that the bacterial pathogens were grown under 11 different stress conditions mimicking the human host environments were integrated with the genome-scale metabolic models of 8 pathogenic species (*Enterococcus faecalis* OG1R, *Escherichia coli* EPEC O127:H6 E2348/69, *Escherichia coli* ETEC H10407, *Escherichia coli* UPEC 536, *Klebsiella pneumoniae* MGH 78578, *Pseudomonas aeruginosa* PAO1, *Staphylococcus aureus* MRSA252, and *Staphylococcus aureus* MSSA476). The resulting reporter metabolites were enriched in multiple metabolic pathways, with cofactor biosynthesis being the most important. The results of this study will serve as a guide for the development of antimicrobial agents as they provide a first insight into potential drug targets.

## Introduction

Antimicrobial resistance is a major global health problem that causes significant morbidity and mortality worldwide. By 2050, this problem could potentially lead to up to 10 million annual deaths worldwide (O’Neill 2014; de Kraker et al. 2016). Due to its impact on human health, the World Health Organization designated antimicrobial resistance as one of the top ten global health threats in 2019 (World Health Organization 2019). Although antimicrobial resistance is widely recognized as a significant global health threat, progress in the discovery and development of new antibiotics has been remarkably unsatisfactory. Several factors, including the lack of interest from major pharmaceutical companies, the rapid emergence and spread of resistance in pathogenic bacteria, and the lack of validated cellular and molecular targets, have contributed to the limited availability of effective antimicrobials for the treatment of resistant infections (Årdal et al. 2018, 2019; Prasad et al. 2022). Since most existing antimicrobials primarily target a narrow spectrum of bacterial functions, such as cell envelope synthesis, protein or nucleic acid synthesis, or folic acid synthesis, there is a clear need for antimicrobials that address unexplored or a broad range of potential targets. One of the major challenges in antimicrobial discovery is the selection of suitable targets, emphasizing the need to pursue molecular targets that are less susceptible to the rapid development of resistance (Silver 2011; Laxminarayan et al. 2013; Cheng et al. 2016).

Bacteria possess protective mechanisms to adapt to challenging and detrimental conditions that trigger both general and specific responses, including responses to DNA damage, acid, heat, cold, starvation, oxidative, and osmotic stress (Dawan and Ahn 2022). These stress-induced regulatory systems are crucial for bacterial survival mechanisms and influence processes such as adaptation, physiological alterations, and virulence potential (Giuliodori et al. 2007). Furthermore, activation of stress responses in bacterial physiology can lead to reduced susceptibility to various antimicrobials by stimulating resistance mechanisms, promoting a resistant lifestyle, and even inducing resistance mutations. Consequently, the activation of bacterial stress responses poses a significant threat to the efficacy and clinical success of antimicrobial treatment. A comprehensive understanding of stress response regulation, particularly in relation to antimicrobial resistance, provides valuable insights for the development of novel and effective therapeutic strategies to combat this problem (Dawan and Ahn 2022).

The cellular response to genetic and environmental changes is often manifested by transcription, translation, and post-translational modifications that lead to changes in gene expression, protein activity, and cellular metabolism. These metabolic adaptations are often initiated by changes in gene expression that are regulated by complex mechanisms orchestrating different metabolic pathways. Therefore, metabolic networks can serve as a framework for mapping the differential expression data of all genes and thus represent the effects of perturbations at the metabolic pathway level by utilizing network connectivity (Patil and Nielsen 2005; Zelezniak et al. 2010). Patil and Neilsen (2005) have developed an algorithm based on hypothesis-driven data analysis to reveal the transcriptional regulatory structure of metabolic networks (Patil and Nielsen 2005). This algorithm allows the identification of ‘reporter metabolites’, i.e. metabolites around which the most significant transcriptional changes occur, together with a set of interconnected genes that show substantial and coordinated responses to genetic or environmental perturbations. Reporter metabolites play a pivotal role in unraveling the intricate web of biochemical pathways underlying various phenotypes. These metabolites, whose abundance dynamically reflects the activity of specific pathways or cellular processes, serve as valuable indicators for understanding biological mechanisms. By monitoring changes in reporter metabolite levels, the metabolic fingerprints associated with different phenotypic traits, such as disease states or responses to environmental stimuli can be deciphered (Montagud et al. 2010; Kori and Arga 2018).

In the current study, the pathway-driven computational approach was used to determine the potential antimicrobial targets based on the transcriptomic data obtained under 11 different stress conditions, including acidic stress, bile stress, hypoxia, low iron, nitrosative stress, nutritional downshift, osmotic stress, oxidative stress, stationary phase, temperature, and virulence inducing conditions for 8 pathogenic species, namely *Escherichia coli* EPEC 0127:H6 E2348/69, *Escherichia coli* ETEC H10407, *Escherichia coli* UPEC 536, *Enterococcus faecalis* OG1RF, *Klebsiella pneumoniae* MGH 78,578, *Pseudomonas aeruginosa* PAO1, *Staphylococcus aureus* MRSA252, and *Staphylococcus aureus* MSSA476 (Avican et al. 2021), as the majority of infections and deaths are attributable to this small group of multidrug-resistant bacteria (Cassini et al. 2019). This systems biology approach will reduce the reliance on labor-intensive, costly, and time-consuming experimental methods, and the results will provide crucial insights for the development of new therapeutic solutions to antimicrobial resistance.

## Materials and methods

### Gene expression datasets

In this study, transcriptome datasets of 8 different bacteria including *Enterococcus faecalis* OG1R (efi), *Escherichia coli* EPEC O127:H6 E2348/69 (ecg), *Escherichia coli* ETEC H10407 (elh), *Escherichia coli* UPEC 536 (ecp), *Klebsiella pneumoniae* MGH 78,578 (kpn), *Pseudomonas aeruginosa* PAO1 (pae), *Staphylococcus aureus* MRSA252 (sar), and *Staphylococcus aureus* MSSA476 (sas) were used. Each bacterium was exposed to 10 infection-relevant stress conditions, including acid stress, bile stress, hypoxia, low iron, nitrosative stress, nutritional downshift, osmotic stress, oxidative stress, stationary phase, and temperature, as well as additional species-specific in vitro virulence-inducing conditions. The libraries were prepared by using three biological replicates of all conditions for each bacterium (Avican et al. 2021). The datasets containing sequencing reads and analyzed data produced in the research are accessible at GEO under the accession number GSE152295.

### Draft model reconstruction for the microorganisms

The draft genome-scale metabolic models (GEMs) of the organisms were reconstructed using the RAVEN Toolbox v2.8.0 (Wang et al. 2018). The reconstruction was performed in MATLAB R2023a (version: 9.14.0.2254940 (R2023a) Update 2). The FASTA files of the protein sequences were obtained from the National Center for Biotechnology Information (NCBI).

The RAVEN toolbox generates the GEMs using metabolic pathway databases, KEGG and MetaCyc, or based on protein homology to an existing template model (Wang et al. 2018). In this study, the GEMs of the microorganisms were reconstructed *de novo* based on the KEGG database. Two draft models were created using the *getKEGGModelForOrganism* function. The first model used the KEGG organism identifier, while the second model queried the proteomes of the microorganisms with Hidden Markov Models (HMMs) trained on prokaryotic sequences with 90% sequence identity. Subsequently, these two models were combined with the *mergeModels* function. The GEMs of the microorganisms were used for further analysis.

### Reporter metabolite analysis

The *Reporter Features* algorithm, which is based on the topology of the metabolic network, was used to identify the reporter metabolites (Patil and Nielsen 2005; Oliveira et al. 2008). First, the algorithm transforms the GEM into a bipartite metabolic graph. Metabolites and enzymes are represented as nodes, while the interactions between them are represented as edges in the metabolic graph. Within this graph, each metabolite node is scored based on the normalized transcriptional response of its neighboring enzymes. By using the p-values of the genes to score the enzyme nodes, the algorithm identifies reporter metabolites, i.e. metabolites surrounded by the most significant transcriptional changes, helping to gain insights into metabolism from the gene expression data. Reporter metabolites with p-values < 0.05 were categorized as statistically significant and used for the enrichment analysis.

### Enrichment analysis of the reporter metabolites

The Metabolites Biological Role-MBRole (v3.0) database (Lopez-Ibanez et al. 2023) was used to find the association of significant reporter metabolites determined under different conditions for different microorganisms. This database provides access to the organism-specific, pre-compiled list of metabolites on KEGG as a reference (Kanehisa and Goto 2000; Kanehisa et al. 2006) and assigns the compounds to metabolic pathways. Pathways that were enriched with a false discovery rate (FDR) < 0.05 were accepted as statistically significant.

## Results

### GEM reconstruction and formation of metabolic graphs

The draft models of the microorganisms were reconstructed by RAVEN toolbox v2.8.0 (Wang et al. 2018), a MATLAB suite primarily designed for the reconstruction of GEMs and the performance of constraint-based analysis, using the protein sequences obtained from NCBI. The draft models based on the KEGG database were used for further analysis (Table 1).

**Table 1:** The summary of the draft GEMs

Microorganisms	Number of Genes	Number of Metabolites	Number of Reactions
*Enterococcus faecalis* OG1R	640	1,391	1,172
*Escherichia coli* EPEC O127:H6 E2348/69	1,149	1,865	1,760
*Escherichia coli* ETEC H10407	1,156	1,908	1,810
*Escherichia coli* UPEC 536	1,174	1,918	1,793
*Klebsiella pneumoniae* MGH 78578	1,348	2,003	1,934
*Pseudomonas aeruginosa* PAO1	1,346	2,050	1,892
*Staphylococcus aureus* MRSA252	642	1,475	1,301
*Staphylococcus aureus* MSSA476	637	1,476	1,305

To enhance the accuracy of the GEMs, spontaneous reactions, which can occur without the need for a catalyst or external energy, were deleted from the draft models. The metabolic graphs were prepared using the instructions of *Reporter Features* (Patil and Nielsen 2005; Oliveira et al. 2008) enabling a detailed classification of the reactions based on KEGG pathways. The classification encompassed a wide array of metabolic processes, categorized into the following pathways: Carbohydrate metabolism, energy metabolism, lipid metabolism, nucleotide metabolism, amino acid metabolism, metabolism of other amino acids, glycan synthesis and metabolism, metabolism of cofactors and vitamins, metabolism of terpenoids and polyketides, biosynthesis of other secondary metabolites, xenobiotics biodegradation and metabolism, translation, and unknown. This comprehensive categorization allowed for a systematic analysis of the metabolic capabilities and potential stress responses of the bacterial species under study.

### Determination of the reporter metabolites and enriched KEGG pathways

The integration of transcriptomic data with the GEMs was a crucial step in identifying reporter metabolites. These metabolites, which exhibited significant alterations under different stress conditions, were pivotal in understanding the stress response mechanisms of the bacteria. RNA-Seq data was integrated into the metabolic graphs using the p-values of gene expression data to determine the reporter metabolites. Reporter metabolites with p-values < 0.05 of 8 bacteria grown under 11 different stress conditions were counted as significant. Numbers of the significant/nonsignificant reporter metabolites of each bacterium are presented in Table 2.

**Table 2:** The number of the significant/nonsignificant reporter metabolites under different conditions (p < 0.05)

Microorganism	Acidic stress	Bile stress	Hypoxia	Low iron	Nitrosative stress	Nutritional downshift	Osmotic stress	Oxidative stress	Stationary phase	Temperature	Virulence inducing conditions
*E. faecalis*	82/995	59/1,018	80/997	97/980	55/1,022	69/1,008	53/1,024	118/959	94/983	69/1,008	64/1,013
*E. coli* EPEC	79/1,567	84/1,562	117/1,529	108/1,538	96/1,550	90/1,556	124/1,522	106/1,540	93/1,553	103/1,543	139/1,507
*E. coli* ETEC	104/1,579	108/1,575	104/1,579	115/1,568	120/1,563	81/1,602	122/1,561	102/1,581	125/1,558	107/1,576	130/1,553
*E. coli* UPEC	145/1,546	147/1,544	97/1,594	124/1,567	138/1,553	127/1,564	112/1,579	90/1,601	127/1,564	154/1,537	100/1,591
*K. pneumoniae*	127/1,654	120/1,661	129/1,652	203/1,578	146/1,635	171/1,610	149/1,632	152/1,629	149/1,632	160/1,621	118/1,663
*P. aeruginosa*	200/1,657	190/1,667	90/1,767	112/1,745	112/1,745	122/1,735	131/1,726	134/1,723	79/1,778	147/1,710	171/1,686
*S. aureus* MRSA	65/1,181	71/1,175	76/1,170	76/1,170	113/1,133	63/1,183	115/1,131	49/1,197	68/1,178	131/1,115	56/1,190
*S. aureus* MSSA	81/1,166	78/1,169	73/1,174	60/1,187	66/1,181	52/1,195	77/1,170	65/1,182	84/1,163	71/1,176	80/1,167

**Fig. 1:**
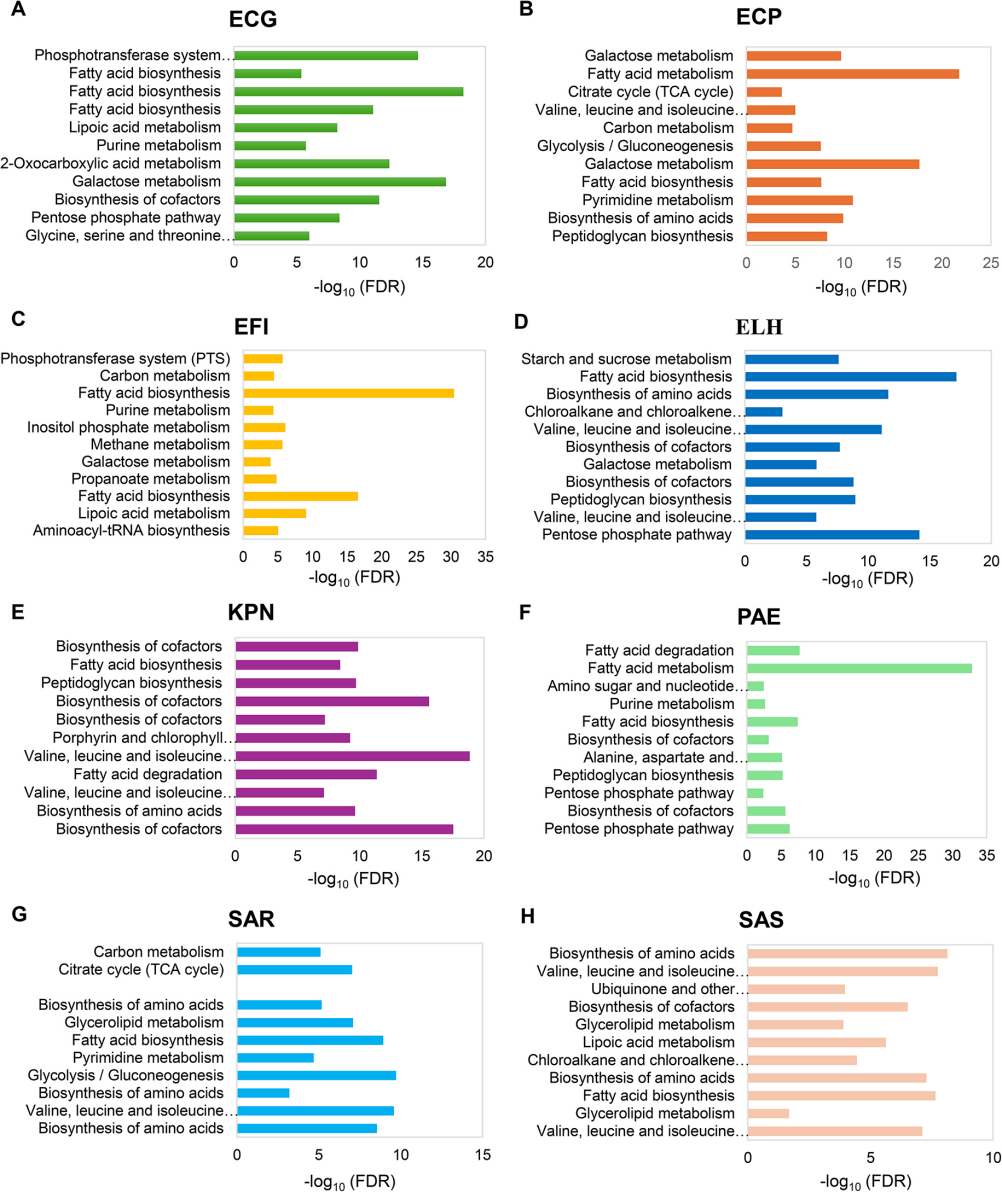
The most enriched metabolic pathways for each bacterial strain under various stress conditions. The pathways are ordered from the top according to the conditions of acidic stress, bile stress, hypoxia, low iron, nitrosative stress, nutritional downshift, osmotic stress, oxidative stress, stationary phase, temperature, and virulence inducing for (**A**) *E. coli* EPEC O127:H6 E2348/69, (**B**) *E. coli* UPEC 536, (**C**) *E. faecalis* OG1R, (**D**) *E. coli* ETEC H10407, (**E**) *K. pneumoniae* MGH 78578, (**F**) *P. aeruginosa* PAO1, (**G**) *S. aureus* MRSA252, and (**H**) *S. aureus* MSSA476

To gain a deeper understanding of the metabolic activities related to significant reporter metabolites, additional analyses were performed using the MBROLE (v3.0) database. The analysis revealed that the identified reporter metabolites were enriched in multiple metabolic pathways. Pathways with FDR < 0.05 were accepted as statistically significant. The most enriched metabolic pathways for each bacterial strain under various stress conditions are given in Fig. 1.

**Fig. 2:**
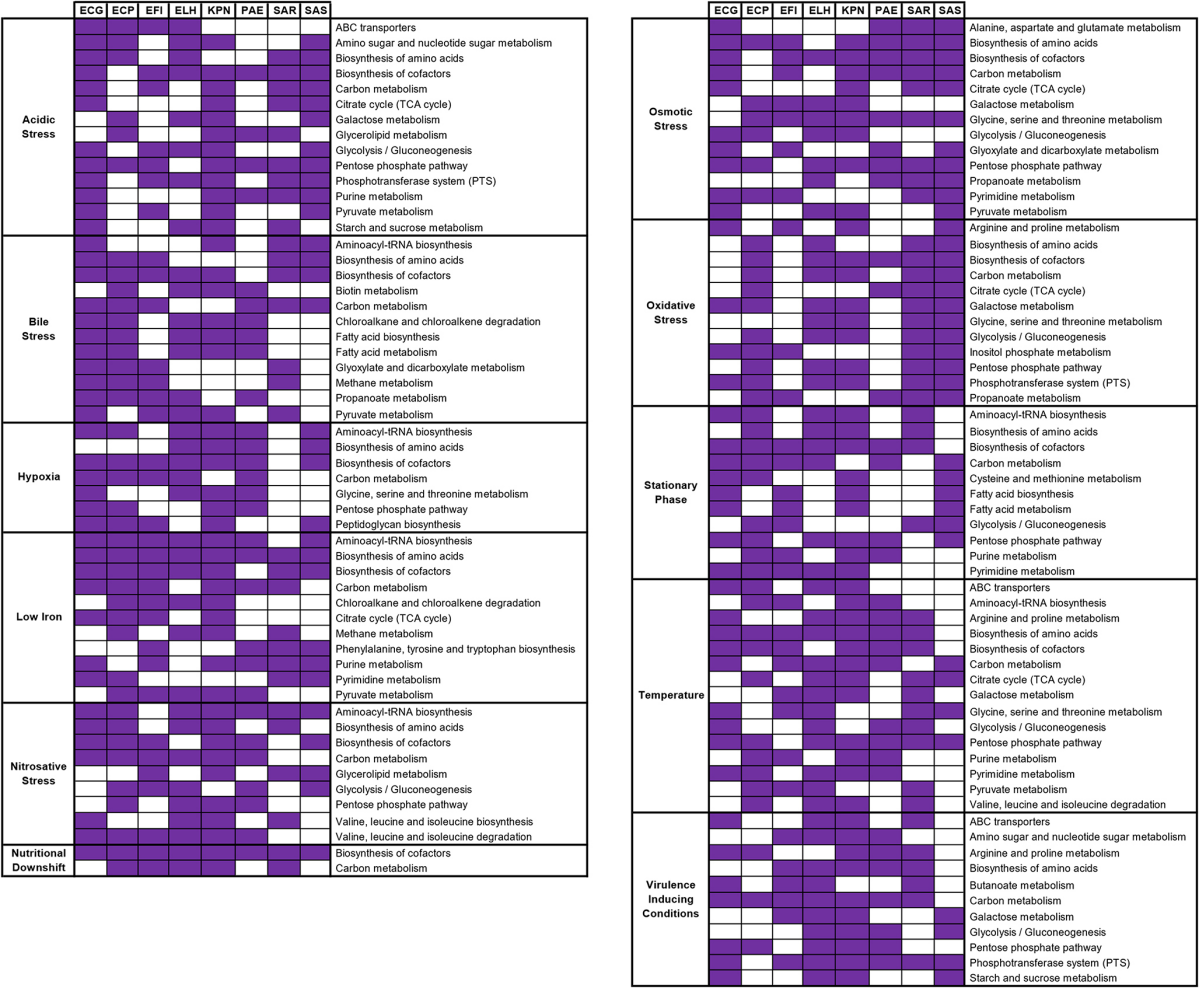
Common enriched pathways of 8 bacteria under 11 different stress conditions

**Fig. 3:**
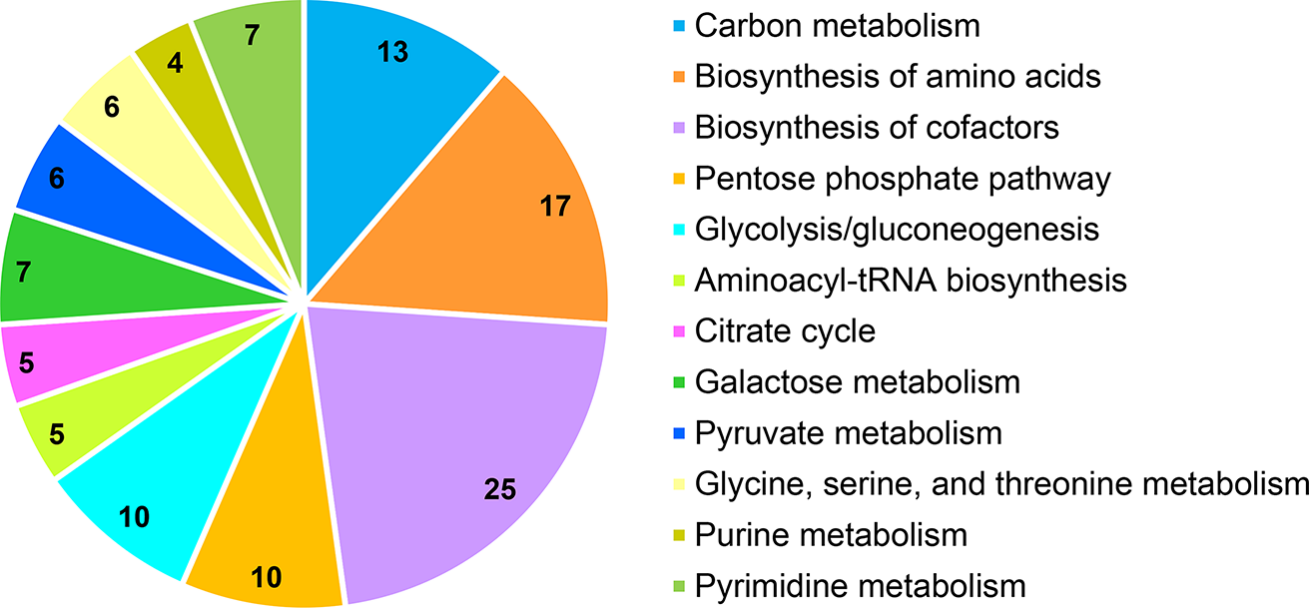
Distribution of common reporter metabolites

The enriched pathways were further detailed for each stress condition to understand whether bacteria respond similarly to different stress conditions. The most common enriched pathways under several stress conditions (number of the stress conditions were given in parentheses) are carbon metabolism (11), biosynthesis of amino acids (10), biosynthesis of cofactors (10), pentose phosphate pathway (8), glycolysis/gluconeogenesis (7), aminoacyl-tRNA biosynthesis (6), citrate cycle (5), galactose metabolism (5), pyruvate metabolism (5), glycine, serine, and threonine metabolism (4), purine metabolism (4), and pyrimidine metabolism (4) (Fig. 2). The most common metabolites involved in these pathways are listed in Table 3. The pathway containing the highest number of reporter metabolites is cofactor biosynthesis, followed by biosynthesis of amino acids as depicted in Fig. 3.

**Table 3:** The most common metabolites involved in the common pathways

Pathways	Common Metabolites
Carbon metabolism	Phosphoenolpyruvate, D-Fructose 6-phosphate, D-Ribose 5-phosphate, 5,10-Methylenetetrahydrofolate, Glycerone, D-Ribulose 5-phosphate, beta-D-Glucose, D-Gluconic acid, alpha-D-Glucose, Isocitrate, 6-Phospho-D-gluconate, alpha-D-Glucose 6-phosphate, beta-D-Fructose 6-phosphate
Biosynthesis of amino acids	Phosphoenolpyruvate, D-Ribose 5-phosphate, 3-Methyl-2-oxobutanoic acid, L-Asparagine, L-Valine, D-Ribulose 5-phosphate, Isocitrate, L-Isoleucine, L-Aspartate 4-semialdehyde, L-Histidinol phosphate, N-(L-Arginino)succinate, (S)-1-Pyrroline-5-carboxylate, (R)-2,3-Dihydroxy-3-methylbutanoate, beta-D-Fructose 6-phosphate, (S)-2-Aceto-2-hydroxybutanoate, (R)-2,3-Dihydroxy-3-methylpentanoate, (S)-2-Acetolactate
Biosynthesis of cofactors	AMP, Phosphoenolpyruvate, UTP, D-Ribose 5-phosphate, 3-Methyl-2-oxobutanoic acid, 5,10-Methylenetetrahydrofolate, L-Valine, D-Ribulose 5-phosphate, Isocitrate, (S)-Dihydroorotate, Retinal, Retinol, alpha-D-Glucose 6-phosphate, Menaquinone, Isochorismate, Orotidine 5’-phosphate, 2-Succinylbenzoate, Dihydrolipoylprotein, 2-Succinylbenzoyl-CoA, beta-D-Fructose 6-phosphate, 3-Aminopropanal, Menaquinol, Molybdopterin, Enzyme N6-(lipoyl)lysine, Enzyme N6-(dihydrolipoyl)lysine
Pentose phosphate pathway	D-Glucose, D-Ribose 5-phosphate, D-Ribose, D-Ribulose 5-phosphate, beta-D-Glucose, D-Gluconic acid, 6-Phospho-D-gluconate, alpha-D-Glucose 6-phosphate, 2-Deoxy-D-ribose 5-phosphate, beta-D-Fructose 6-phosphate
Glycolysis/gluconeogenesis	D-Glucose, Phosphoenolpyruvate, beta-D-Glucose, alpha-D-Glucose, Ethanol, alpha-D-Glucose 6-phosphate, beta-D-Fructose 6-phosphate, Enzyme N6-(lipoyl)lysine, Enzyme N6-(dihydrolipoyl)lysine, Enzyme N6-(S-acetyldihydrolipoyl)lysine
Aminoacyl-tRNA biosynthesis	L-Asparagine, L-Valine, L-Isoleucine, L-Lysyl-tRNA, L-Seryl-tRNA(Sec)
Citrate cycle	Phosphoenolpyruvate, Isocitrate, Enzyme N6-(lipoyl)lysine, Enzyme N6-(dihydrolipoyl)lysine, Enzyme N6-(S-acetyldihydrolipoyl)lysine
Galactose metabolism	D-Glucose, UDP-alpha-D-galactose, D-Fructose 6-phosphate, Glycerol, alpha-D-Glucose, alpha-D-Galactose 1-phosphate, alpha-D-Glucose 6-phosphate
Pyruvate metabolism	Phosphoenolpyruvate, Ethanol, Methylglyoxal, Enzyme N6-(lipoyl)lysine, Enzyme N6-(dihydrolipoyl)lysine, Enzyme N6-(S-acetyldihydrolipoyl)lysine
Glycine, serine, and threonine metabolism	5,10-Methylenetetrahydrofolate, L-Aspartate 4-semialdehyde, Methylglyoxal, [Protein]-S8-aminomethyldihydrolipoyllysine, Dihydrolipoylprotein, L-2-Amino-3-oxobutanoic acid
Purine metabolism	AMP, D-Ribose 5-phosphate, dGDP
Pyrimidine metabolism	UTP, Uracil, Uridine, (S)-Dihydroorotate, Cytosine, Orotidine 5’-phosphate, Methylmalonate

## Discussion

The main causes of antimicrobial resistance are the improper and excessive use of antibiotics, often due to presumptive treatment and the inappropriate use of broad-spectrum antibiotics with inconsistent dosing (Aly and Balkhy 2012; Van Goethem et al. 2024). The declining antimicrobial efficacy of many existing drugs combined with the increasing prevalence of drug-resistant strains poses a major therapeutic challenge. Research is therefore ongoing to identify new drugs, novel targets, the use of synergistic drug combinations, and the repositioning of the existing drugs (Yaneja and Kaur 2016; Sun et al. 2016).

Exposure to various stressors, including nutrient stress, oxidative/nitrosative stress, envelope stress, heat stress, and ribosomal stress, has a notable impact on bacterial susceptibility to a range of antimicrobial agents. This influence results from triggering stress responses, which can lead to the recruitment of resistance mechanisms or physiological changes that impair the efficacy of antimicrobial agents. As stress responses are an essential component of antimicrobial resistance, even multidrug resistance, they should be considered as potential targets for therapeutic intervention (Poole 2012).

The regulation of bacterial stress responses leads to alterations in gene expression, protein activity, and cellular metabolism (Dawan and Ahn 2022). In this context, the study of the pathogen’s entire metabolism at the genome-scale can provide a comprehensive understanding for the identification of more effective drug targets and a deeper understanding of the pathogen’s characteristics. GEMs are often used to decipher pathogen and host metabolism as they provide a holistic, system-wide perspective (Cesur et al. 2020). *In silico* analyses based on GEMs can significantly narrow down potential drug targets.

In the current study, a systems biology approach was applied to evaluate the potential antibiotic targets. For this purpose, gene expression data measured under different stress conditions (Avican et al. 2021), reconstructed GEMs of the selected microorganisms, and a pathway-driven computational algorithm (Patil and Nielsen 2005) were used to explore the transcriptional differences related to metabolism under resistance-related stress conditions. The GEMs were transformed into bipartite metabolic graphs. Within this graph, each metabolite node is scored based on the normalized transcriptional response of its neighboring enzymes. By using the p-values of genes as input for scoring the enzyme nodes, the algorithm identifies so-called ‘reporter metabolites’. These are metabolites around which the most significant transcriptional changes are observed. As seen in Table 2, over 1,000 reporter metabolites were obtained after the analysis. By focusing on the significant ones with p-values < 0.05, the time-consuming phase for target identification was eliminated. Pathway enrichment was then used to link the resulting significant reporter metabolites to the organism-specific KEGG pathways.

The most common enriched pathways between the microorganisms under 11 different stress conditions were determined as carbon metabolism, biosynthesis of amino acids, biosynthesis of cofactors, pentose phosphate pathway, glycolysis/gluconeogenesis, aminoacyl-tRNA biosynthesis, citrate cycle, galactose metabolism, pyruvate metabolism, glycine, serine, and threonine metabolism, purine metabolism, and pyrimidine metabolism. Among these, carbon metabolism, biosynthesis of amino acids, biosynthesis of cofactors, aminoacyl-tRNA biosynthesis, and pyruvate metabolism are common pathways in all Gram-positives while carbon metabolism, biosynthesis of amino acids, biosynthesis of cofactors, pentose phosphate pathway, glycolysis/gluconeogenesis, and aminoacyl-tRNA biosynthesis are common in all Gram-negatives. Additionally, ABC transporters, glycine, serine, and threonine metabolism, and purine metabolism were enriched in many Gram-negative bacteria.

The intricate interplay of metabolic pathways highlights potential targets for the development of novel antimicrobial therapies. Understanding these biochemical mechanisms provides valuable insights into developing targeted antimicrobial therapies that disrupt these critical pathways and enhance the efficacy of existing treatments. Carbon metabolism, including glycolysis and the pentose phosphate pathway, is crucial for energy production and provides precursor metabolites for biosynthesis, ensuring sufficient ATP and reducing power to counteract oxidative stress and support repair mechanisms. The decline in energy metabolism observed leads to a reduction in reactive oxygen species production, which enhances antimicrobial resistance. As a result, this limits the frequency of mutations in essential metabolic genes, directly leading to the development of antibiotic resistance. Enhanced carbon metabolism also increases efflux pump activity, energy-dependent repair mechanisms, and biofilm formation, contributing to resistance (Lopatkin et al. 2021; Kok et al. 2022; Tong and Brown 2023) Similarly, glycolysis and gluconeogenesis pathways ensure continuous ATP supply and metabolic flexibility under nutrient limitation and stress conditions, powering efflux pumps and other energy-dependent resistance mechanisms. The citrate cycle (TCA cycle) ensures adequate energy production and supplies biosynthetic intermediates for repair and adaptation, supporting resistance mechanisms and the synthesis of resistance proteins. Pyruvate metabolism maintains energy and precursor supply, supporting cellular repair mechanisms and resistance factor synthesis (Tong and Brown 2023). The pentose phosphate pathway, producing NADPH and ribose-5-phosphate, is critical for antioxidant defense and nucleotide biosynthesis, supporting DNA repair and replication during stress, and contributing to resistance by maintaining redox balance and repairing antibiotic-induced DNA damage (Stincone et al. 2015). The purine and pyrimidine metabolism ensures a continuous supply of nucleotides for DNA repair and replication, supporting the repair of antibiotic-induced DNA damage and enabling the rapid production of resistance genes (Lopatkin and Yang 2021). The biosynthesis of amino acids supports protein synthesis that contributes the resistance by decreasing the concentrations of antimicrobials or modifying them. Upregulation ensures a continuous supply of amino acids for the porins/efflux proteins and enzymes, aiding in cellular recovery and adaptation, and contributing to resistance (Idrees et al. 2020). Aminoacyl-tRNA biosynthesis supports rapid protein synthesis for stress adaptation. Additionally, they have a role in the biosynthesis of the bacterial cell envelope that affects how the cell interacts with antibiotics and antimicrobial peptides (RajBhandary and Söll 2008; Chopra and Reader 2015). Lastly, the biosynthesis of cofactors is essential for maintaining redox balance and supporting enzyme activity (Sun et al. 2023).

One promising aspect for antibacterial discovery initiatives lies in the fact that many amino acid and vitamin biosynthetic pathways are highly conserved in bacteria and have no human homologs, apart from the glutamine, glycine, proline, serine, nucleotide, and folate pathways, which are at least partially present in humans. Importantly, there is promising potential for antibiotic discovery, as the ability to selectively target bacterial or human enzymes has been demonstrated in nucleotide (Marcinkeviciene et al. 2000), amino acid (Amorim Franco and Blanchard 2017), and cofactor biosynthesis (Wróbel et al. 2019).

### Biosynthesis of cofactors- a promising antibiotic target

Cofactors play a vital role in maintaining the redox balance in cells and are required for cell-based biotransformations. They are involved in almost all enzymatic functions in living cells (Sun et al. 2023). In the current study, cofactor biosynthesis is one of the main pathways affected by all stress conditions except virulence induction. All reporter metabolites were analyzed for each bacterium in detail (Table 4).

**Table 4:** Common reporter metabolites in cofactor biosynthesis under different stress conditions

Microorganism	Reporter Metabolite
*E. faecalis*	Riboflavin, Reduced riboflavin
*E. coli* EPEC	Molybdopterin, Menaquinone, Menaquinol, Glutathione, Tetrahydrofolate, 5,10-Methylenetetrahydrofolate
*E. coli* ETEC	Adenylated molybdopterin, Menaquinone, Menaquinol, Pyrrolo-quinoline quinone, Reduced pyrroloquinoline-quinone, 5,10-Methylenetetrahydrofolate, Dihydropteroate
*E. coli* UPEC	Molybdopterin, Precursor Z, Menaquinone, Menaquinol
*K. pneumoniae*	Precorrin 2, Precorrin 3 A, Cobyrinate, Sirohydrochlorin, Precorrin 6Y, Precorrin 6Y, Hydrogenobyrinate, Precorrin 3B, Precorrin 4, Precorrin 8X, Precorrin 5, Cob(II)yrinate a, c diamide, Adenosyl cobyrinate a, c diamide, Adenosyl cobyrinate hexaamide, Adenosyl cobinamide, Adenosyl cobinamide phosphate, Cobalt-sirohydrochlorin, Cobalt-precorrin 3, Cobalt-precorrin 4, Cobalt-precorrin 6, Cobalt-dihydro-precorrin 6, Cobalt-precorrin 8, Cobalt-precorrin 5 A, Cobalt-precorrin 5B, Cobalt-factor III
*P. aeruginosa*	Coproporphyrinogen III, Cobalt-precorrin 4
*S. aureus* MRSA	(S)-Dihydroorotate
*S. aureus* MSSA	Menaquinone, Phylloquinone, Phylloquinol

In *E. faecalis*, riboflavin and reduced riboflavin were among the common metabolites for cofactor biosynthesis. Riboflavin contains a crucial prosthetic group consisting of two important cofactors, the flavin mononucleotide (FMN) and the flavin adenine dinucleotide (FAD). These coenzymes are essential for various oxidation-reduction reactions and act as essential components for oxidases, reductases, and dehydrogenases. They play a key role in the processes of energy metabolism. Higher animals, including humans, usually acquire riboflavin through their daily diet, as they lack an internal mechanism for the endogenous production of riboflavin (Saedisomeolia and Ashoori 2018). Therefore, riboflavin biosynthetic pathways in microbes may represent a promising target for drug development, especially for pathogenic microbes that rely on internal biosynthesis, as suggested by Farah et al. (Farah et al. 2022). Additionally, Gerdes et al. identified some antimicrobial drug targets using genetic footprinting in *E. coli* followed by metabolic context analysis of essential gene orthologs in various species including *P. aureginosa* and *S. aureus*. In their study, a conserved *de novo* riboflavin biosynthetic pathway was reported in the majority of bacteria, which is not present in humans. In this *de novo* pathway, all the genes were identified as essential in *E. coli* which makes many of the gene products as potential antimicrobial drug targets. Specifically, FAD synthase was underlined as an attractive target (Gerdes et al. 2002). GTP cyclohydrolase II (GCH II), lumazine synthase (LS), riboflavin synthase (RFS), and the FMN riboswitch are also reported to be good drug targets. By inhibiting these enzymes and the FMN riboswitch, disrupting flavin-dependent metabolic pathways, such as ATP biosynthesis, redox reactions, and fatty acid metabolism in pathogens, can be achieved without interfering with the metabolic activities of the human host (Jaroensuk et al. 2023).

Molybdopterin is a common reporter metabolite in *E. coli* strains. It is synthesized from Precursor Z and its complex with the Mo atom is required for the activity of all mononuclear molybdoenzymes. The Mo-molybdopterin biosynthesis enzymes have been reported to be crucial for the virulence of various pathogenic bacteria, including *E. coli* and *Mycobacterium tuberculosis* (Zhong et al. 2020). Mutations in the Mo-molybdopterin synthesis pathway completely inhibit cofactor formation, which in turn eliminates the activity of all Mo enzymes and leads to a pleiotropic phenotype. Defects in the *moaA* gene in *Enterobacter cloacae*, *Klebsiella oxytoca* (Hughes et al. 2017), and *Salmonella typhi* (Contreras et al. 1997), *moaA1-D1* locus (Levillain et al. 2017), *moaC1* (Dutta et al. 2010), *moaD1* (Brodin et al. 2010), *moaX* (Rosas-Magallanes et al. 2007), and *moeB1 *(MacGurn and Cox 2007) in *M. tuberculosis*, *PA1006* in *P. aeruginosa* (Filiatrault et al. 2013), and *mobAB* in *E. coli * (Zhang et al. 2019) have been shown to cause significantly reduced fitness or virulence. Although, there are homologous genes with humans, the genes that are unique to prokaryotes could be evaluated as promising targets for the development of new antibacterial agents.

Menaquinones (the vitamin K2-reduced form is menaquinol) are essential components of cell membranes and play a crucial role in the electron transport chain, virulence, endospore formation, and the formation of cytochromes in many anaerobic bacteria, mycobacteria, and Gram-positive bacteria (Li et al. 2010). In *E. coli* strains, menaquinone and menaquinol are notable as significant reporter metabolites. Likewise, in *S. aureus* MSSA, common reporter metabolites include phylloquinol and phylloquinone, which are derived from menaquinone synthesis, as well as menaquinone itself (Table 4). The bacterial menaquinone synthesis starts from chorismate and is catalyzed by the enzymes MenF, MenD, MenH, MEnC, MenE, MEnB, MenI, MenA, and MenG (Paudel et al. 2016). In contrast to mammals, which use ubiquinone instead of menaquinone for respiration, humans cannot produce menaquinone themselves and must absorb it through food or intestinal flora. Consequently, the biosynthetic pathway of menaquinone represents a promising target for the development of new antimicrobial agents for the treatment of infections caused by pathogens that rely on menaquinone (Paudel et al. 2016; Belete 2019). Several research studies have identified inhibitor molecules that target MenD (Fang et al. 2010), MenE (Lu et al. 2008, 2012; Tian et al. 2008), MenB (Li et al. 2010; Matarlo et al. 2016), MenA (Dhiman et al. 2009; Debnath et al. 2012; Choi et al. 2016; Berube et al. 2019), and MenG (Sukheja et al. 2017; Macsics et al. 2020; Pujari et al. 2022) in different bacteria. More importantly, Hamamoto et al. reported the first antibiotic Lysocin E, a cyclic lipopeptide produced by *Lysobacter sp.* RH2180-5, which directly interacts with menaquinone (Hamamoto et al. 2014).

Tetrahydrofolate and its derivatives serve as important cofactors in one-carbon metabolism. While plants and numerous microorganisms synthesize folate coenzymes internally, vertebrates are completely dependent on external nutritional sources for these compounds (Illarionova et al. 2002). The folate biosynthetic pathway is a known target for antimicrobial treatments. Sulfonamides, the first synthetic antimicrobial drugs with a broad spectrum of activity, function by inhibiting dihydropteroate synthase, the penultimate enzyme of the dihydrofolate biosynthetic pathway. Trimethoprim also acts against various bacterial pathogens by inhibiting dihydrofolate reductase (Bourne 2014). Since there are already antimicrobial agents that target the enzymes in this pathway, new potential antimicrobial agents could inhibit the new or existing targets that are not homologous in humans.

Specific attention should be drawn to the reporter metabolites in *K.pneumoniae*.

### Porphyrin metabolism in *K. pneumoniae*

**Fig. 4:**
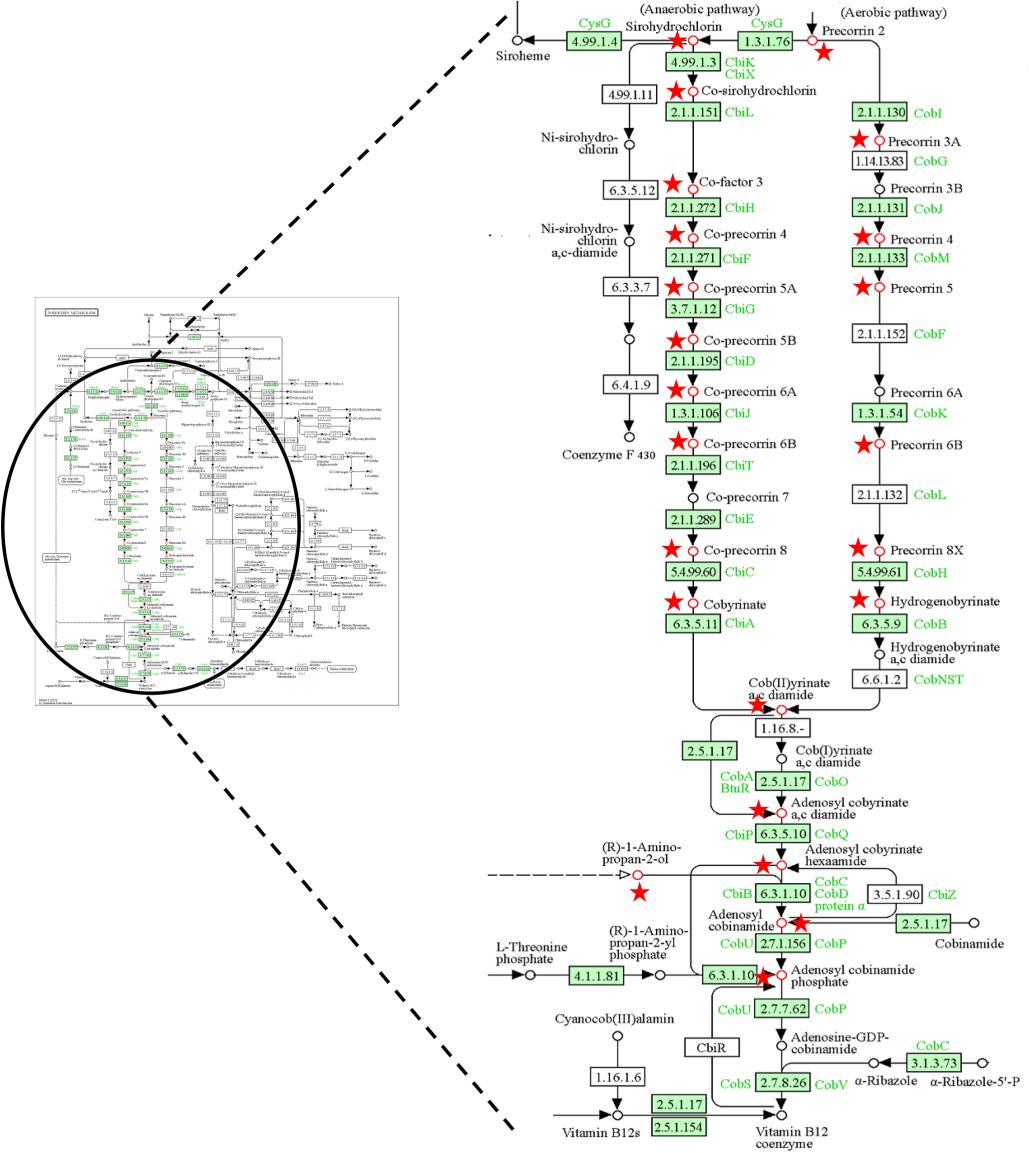
Significant reporter metabolites in *K. pneumoniae* under different stress conditions. The pathway was retrieved from the KEGG Database (Kanehisa et al. 2023)

The reporter metabolites for *K. pneumoniae* were clustered in a specific pathway as shown in Fig. 4. They indicated that there is a significant regulation in porphyrin metabolism, especially in the biosynthesis of cobalamin (vitamin B12). In nature, cobalamin is only synthesized by microorganisms such as *Klebsiella*, *Salmonella*, and related bacteria, whereas humans only absorb cobalamin exclusively from dietary sources. These microorganisms produce cobalamin as an essential cofactor for enzymatic reactions involved in the synthesis of branched-chain acids and provide oxidizing equivalents for growth in anaerobic environments (Xu and Grissom 1999).

Cobalamin biosynthesis was considered a possible target for the development of the drugs in *M. tuberculosis* (Gopinath et al. 2013). Additionally, CobM (precorrin-4 C(11)-methyltransferase) enzyme involved in the cobalamin biosynthesis of *Corynebacterium pseudotuberculosis*, the agent of caseous lymphadenitis, was characterized and proposed as a candidate drug target to reduce the pathogen’s virulence in its hosts (Peinado et al. 2019). The effects of colistin treatment on cobalamin biosynthesis were reported in a study by Sun et al. (Sun et al. 2020). The expression of 14 proteins involved in porphyrin and chlorophyll metabolism, which are mainly enriched in the cobalamin biosynthetic pathways from precorrin-2 to cobyrinic acid-a, c-diamide, was found to be lower in the polymyxin-resistant mutant than in the wild type. Accordingly, porphyrin and chlorophyll metabolism, which represents the biosynthesis of cobalamin, was probably downregulated in the cells treated with a high concentration of colistin.

There is growing interest in research into riboswitches, as they are considered to be optimal candidates for future antibiotics. This is due to their regulatory role and their prevalence in bacteria. Some currently used drugs have been found to work via this mechanism of action (Blount and Breaker 2006). Palou-Mir et al. characterized the cobalamin-dependent *btuB* riboswitch of *K. pneumoniae* (Palou-Mir et al. 2016) and later proposed this riboswitch as a target for new antibiotics (Palou Mir 2019). Recently, a study was conducted by Lee et al. using a combined antibacterial synergy approach and the ANNOgesic tool to identify novel targets within the gene networks of multidrug-resistant *K. pneumoniae* (Lee et al. 2024). Cells were treated with a colistin-chemical #3 (K56_Co_Che) combination. It was emphasized that the cobalamin riboswitch has potential for various applications due to its specificity for bacteria.

The findings from these studies suggest that cobalamin biosynthesis could be a promising target for antimicrobial development.

## Conclusions

New and innovative approaches are essential to confront the imminent threat posed by resistant pathogens and to prevent the emergence of infections that cannot be treated. Unconventional targets, which are uniquely significant during infections and tractable to high-throughput drug discovery methods, offer considerable potential for groundbreaking advancements in antibiotic research. In the current study, reporter metabolites were determined for 8 microorganisms under 11 different stress conditions. The responses under these stress conditions gave clues about the bacterial survival mechanisms, adaptation, physiological alterations, virulence potential, and antibiotic resistance. Then, significant reporter metabolites were placed in several pathways by the enrichment analysis. The common enriched pathways between the microorganisms were carbon metabolism, biosynthesis of amino acids, biosynthesis of cofactors, pentose phosphate pathway, glycolysis/gluconeogenesis, aminoacyl-tRNA biosynthesis, citrate cycle, galactose metabolism, pyruvate metabolism, glycine, serine, and threonine metabolism, purine metabolism, and pyrimidine metabolism while the biosynthesis of cofactors is the most common one among others. One of the most important off-target effects of antimicrobial drug development is the host similarities. Inhibitors targeting the bacterial pathways might also affect similar pathways in host cells, leading to cytotoxicity. But, since the biosynthesis pathways of many cofactors, especially vitamins, are well conserved across bacteria and have no human homologs, they show promise for antimicrobial discovery efforts. Overall, while the findings of the current study present potential targets for antimicrobial discovery, further efforts are essential to fully validate these findings experimentally.

## Data Availability

Large-scale differential expression analysis data and read count tables were requested from Dr. Kemal Avican. The datasets are accessible at GEO under the accession number GSE152295.
